# Is Centralisation of Cancer Services Associated With Under‐Treatment of Patients With High‐Risk Prostate Cancer?—A National Population‐Based Study

**DOI:** 10.1002/cam4.70403

**Published:** 2024-11-11

**Authors:** Lu Han, Emily Mayne, Joanna Dodkins, Richard Sullivan, Adrian Cook, Matthew Parry, Julie Nossiter, Thomas E. Cowling, Alison Tree, Noel Clarke, Jan van der Meulen, Ajay Aggarwal

**Affiliations:** ^1^ Faculty of Public Health and Policy The London School of Hygiene and Tropical Medicine London UK; ^2^ Clinical Effectiveness Unit Royal College of Surgeons of England London UK; ^3^ Institute of Cancer Policy–King's College London London UK; ^4^ Department of Urology University College London London UK; ^5^ Department of Radiotherapy The Royal Marsden Hospital London UK; ^6^ Department of Urology The Christie NHS Trust London UK; ^7^ Department of Oncology Guy's & St Thomas' NHS Trust London UK

**Keywords:** centralisation, equity, radiotherapy, surgery, travel times

## Abstract

**Background:**

Centralising prostate cancer surgical and radiotherapy services, requires some patients to travel longer to access treatment, but its impact on actual treatment utilisation and outcomes is unknown.

**Methods:**

Using national cancer registry records linked to administrative hospital data, we identified all patients with high risk and locally advanced prostate cancer diagnosed between 1 April 2019 and 31 March 2020 in the English National Health Service (*n* = 15,971). Estimated travel times from the patient residential areas to the nearest hospital providing surgery or radiotherapy were estimated for journeys by car and by public transport. Multivariable logistic regression was used to model relationships between travel time and receipt of care with adjustment for patient characteristics.

**Results:**

10,693 (67%) men received radical surgery or radiotherapy (RT) within 12 months of diagnosis. Average travel time to the nearest hospital providing prostatectomy or RT was 23.2 min by private car and 58.2 min by public transport. We found no association between travel time, either by car or public transport and the likelihood of receiving curative treatment. Patients living in the most socially deprived areas, those aged over 70, those with two or more comorbidities, and those of black ethnic origin, were less likely to receive curative treatment (*p*& =& 0.001 for all associations).

**Conclusions:**

The current configuration of national prostate cancer services is not associated with the likelihood of receiving curative treatment. Further increases in capacity will unlikely improve utilisation rates beyond addressing sociodemographic barriers.

## 1 Introduction

Centralisation of cancer services to high‐volume hospitals has been undertaken in many countries based on evidence that centres treating complex cancers in higher volume facilities delivers better patient outcomes for both radiotherapy (RT) and surgical services [1, 2, 3].

However, the potential benefits of service centralisation to improve the quality of care needs to be balanced against an increase in travel times for some patients [4, 5, 6]. In the English NHS, where cancer patients are able to choose cancer hospitals, younger, fitter and more affluent patients travel further for cancer treatments [7, 8]. The increased travel times resulting from centralisation may therefore increase inequalities in access for particular disadvantaged patient groups given their lower willingness to travel. As a result, there is increasing pressure to build new RT centres, specifically, given concerns regarding limited access. However, the impact on utilisation and outcomes on a national level has had limited empirical investigation.

Over the last two decades, prostate cancer surgical services have been centralised into fewer high‐volume centres in the English NHS (in 2024 there were 50 centres across a population of 55 million (65 centres in 2010)), with less than half of these hospitals delivering both prostatectomy and RT [9, 10]. Patients receiving either modality, those living in deprived rural areas face the longest travel times, whilst affluent urban residents have the shortest travel times to their treating centre, leading to calls to increase the capacity of cancer services, particularly in rural areas [11]. For patients with high‐risk or locally advanced disease, surgical or RT treatment is well established as curative treatment [9]. However, national audits in the UK have found that on average since 2015, 35% of men do not receive any radical treatment for their prostate cancer, with substantial variation in utilisation rates across hospitals (50%–90%) [12].

In this national population‐based study in England, we analysed whether travel time to cancer services is associated with undertreatment in patients with high‐risk and locally advanced prostate cancer. We also explore whether travel time within this centralised system is particularly burdensome for older, more comorbid and more socially deprived patient groups.

## 2 Methods

### 2.1 Study Population

English Cancer Registry data were used to identify all men newly diagnosed with high‐risk or locally advanced prostate cancer between 1 April 2019 and 31 March 2020 using the International Classification of Diseases 10th Edition (ICD‐10) code C61 [13].

This database was linked at patient‐level with two routine databases: Hospital Episode Statistics (HES) and The National Radiotherapy Data Set (RTDS). HES is an administrative hospital database in the English NHS, which is a source of surgery‐specific information about operation type and date based on procedure codes [14]. RTDS is a national database containing standardised data, including radiotherapy provider code, date of RT treatment and treatment modality, from all NHS providers of RT services in England [15].

Prostate cancer risk was based on TNM stage [16], Gleason score and PSA level (hereafter referred to as ‘cancer characteristics’), according to a modified D'Amico risk stratification algorithm developed previously by the NPCA [13]. The final cohort for analysis included 15,971 men with high‐risk or locally advanced prostate cancer diagnosed at 123 English hospitals (Figure S1).

### 2.2 Patient Characteristics

English Cancer Registry data were used to identify the diagnosing hospital, date of diagnosis, cancer characteristics, age at diagnosis and ethnicity for each man. Cancer characteristics were used to stratify disease status and provide baseline information. Age at diagnosis was measured in 4 age bands: < 70, 70–74, 75–79 and ≥ 80 years. Men were categorised into ethnic groups comprising White, Asian, Black, Mixed, Other and Not stated/Missing. HES provided information on comorbidities and socioeconomic status. The Royal College of Surgeons (RCS) Charlson score was used to identify comorbid conditions captured in the HES record within 1 year before diagnosis [17]. Socioeconomic deprivation status was determined for patients from the English 2019 Index of Multiple Deprivation (IMD) based on their area of residence, grouped according to quintiles of the national distribution [18]. Patients residential areas were classified as ‘rural’, ‘urban (outside London)’ or ‘London’ using the Office for National Statistics (ONS) rural–urban classification [19].

### 2.3 Travel Time

The location of patient residence was represented by the population‐weighted centroids of their Lower Layer Super Output Areas (LSOAs). There are 32,844 LSOAs in England, defined as small geographic areas that on average include 1500 residents or 650 households. Travel time from patients to hospitals was estimated using a geographic information system, ArcGIS, by inputting patient's LSOAs and the full postcodes of hospitals. Travel time by car was defined as the fastest route (in minutes) using the Ordnance Survey Master Map Highways Network. Travel time by public transport was estimated using the National Public Transport Nodes (NAPTAN) 2019 dataset which includes all public transport access points, i.e. anywhere one can get on or off public transport (including bus, rail, tram, metro and underground). We estimated travel time to the nearest hospital providing prostatectomy or RT for each patient separately by car and public transport. Travel time by car was then categorised in 15‐min bands: ≤ 15, 15–30, 30–45, 45–60 and > 60 min. Travel times by public transport are longer than those by private car and were classified as ≤ 30, 31–60, 61–90, 91–120 and > 120 min. A total of 374 patients lived in LSOAs with missing public transport time or with incomplete routes.

### 2.4 Outcome Variable

We created a binary variable to indicate those patients who underwent radical treatment (defined as surgery, radiotherapy, or both) within 12 months of diagnosis. The OPCS Classification of Interventions and Procedures (OPCS‐4) code ‘M61’ was used to identify men who underwent a radical prostatectomy and their operation date in the HES database [20]. The RTDS data item ‘treatment modality’ was used to select men undergoing RT. Treatment doses included 74–78 Gy to the prostate in 37–39 fractions over 7.5 weeks and 60 Gy in 20 fractions over 4 weeks as per national guidance at this time [21].

### 2.5 Statistical Analysis

We applied multilevel generalised linear regression models with a random intercept by diagnosing hospitals, to estimate associations between receiving radical local treatment, patient characteristics and travel time. We report unadjusted and adjusted risk ratios by specifying the Poisson distribution for the residuals and a log link function, considering outcome events are common (> 10%). Robust standard errors were obtained to account for potential correlations between residuals. With the adjusted model, we further investigated whether the associations of patient characteristics differ according to travel time categories by including interaction terms. The Wald test was performed for the statistical significance of the interaction. Patients with missing data in ethnicity (7.6%) and public travel time (2.3%) were included in our regression models by creating a separate category, as the missing mechanism was likely to be systematic rather than random.

## 3 Results

### 3.1 Patient Characteristics

**FIGURE 1:**
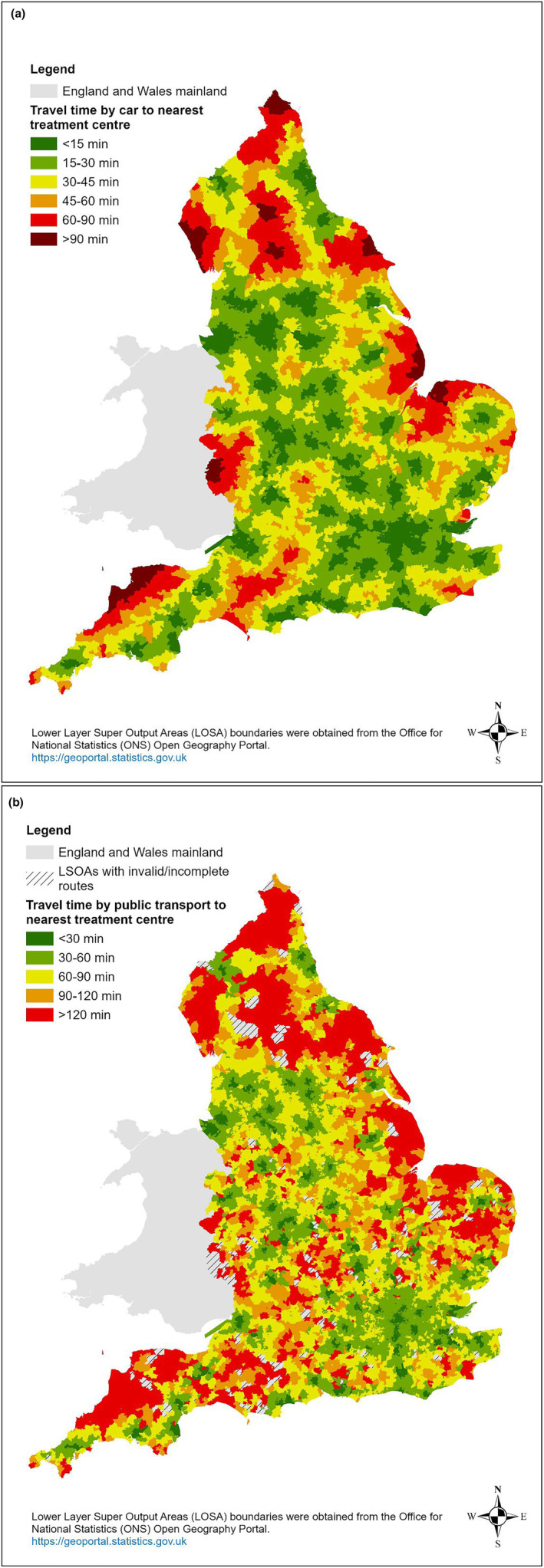
(a) Travel time to the nearest treatment centre (surgery or radiotherapy)–by car. (b) Travel time to the nearest treatment centre (surgery or radiotherapy)–by public transport.

15,971 men were diagnosed with high‐risk or locally advanced prostate cancer between 1 April 2019 and 31 March 2020 (Table 1). The mean age of these patients was 71.1 years (SD = 8.3) and the majority were of White ethnicity (86.2%). Over half (51.2%) lived in socioeconomically deprived areas (IMD 2019 quintiles 3–5), 26% lived in a rural residence and 14.1% had at least one existing comorbidity prior to the diagnosis of cancer. Across all patients, the median travel time to the nearest hospital providing prostatectomy or RT was 18.0 min (IQR: 10.7–29.6) by private car and 53 min (IQR: 37–71) by public transport. Figure 1a,b demonstrate regions in England with the longest travel time to a treating centre by car or public transport.

**TABLE 1:** Patient characteristics.

	Total		Received surgery or RT		Without treatment	
	*n*	%^a^	*n*	%^b^	*n*	%^b^
Number of patients	15,971	100	10,693	67.0	5278	33.1
Age at diagnosis						
Mean (SD)	71.1 (8.3)		68.9 (7.1)		75.5 (8.9)	
< 70	6423	40.2	5205	81.0	1218	19.0
70–74	4010	25.1	3048	76.0	962	24.0
75–79	3234	20.3	2013	62.2	1221	37.8
≥ 80	2304	14.4	427	18.5	1877	81.5
Ethnicity						
White	13,772	86.2	9244	67.1	4528	32.9
Asian	267	1.7	189	70.8	78	29.2
Black	417	2.6	246	59.0	171	41.0
Mixed	73	0.5	45	61.6	28	38.4
Other	226	1.4	141	62.4	85	37.6
Not stated/Missing	1216	7.6	828	68.1	388	31.9
Socio‐economic status (IMD 2019 in national quintiles)						
1 (least deprived)	3818	23.9	2648	69.4	1170	30.6
2	3982	24.9	2695	67.7	1287	32.3
3	3420	21.4	2309	67.5	1111	32.5
4	2660	16.7	1706	64.1	954	35.9
5 (most deprived)	2091	13.1	1335	63.9	756	36.2
RCS Charlson comorbidity score						
0	13,721	85.9	9435	68.8	4286	31.2
1	1623	10.2	974	60.0	649	40.0
2+	627	3.9	284	45.3	343	54.7
Rurality of residence						
Rural	4155	26.0	2839	68.3	1316	31.7
Urban (non‐London)	10,345	64.8	6918	66.9	3427	33.1
London	1471	9.2	936	63.6	535	36.4
Time to nearest surgery/RT centre by car						
Mean (SD)	23.2 (18.6)		23.1 (18.3)		23.3 (19.2)	
Median (IQR)	18.0 (10.7–29.6)		18.1 (10.7–29.7)		17.9 (10.6–29.4)	
< 15 mins	6555	41.0	4373	66.7	2182	33.3
15–30 mins	5514	34.5	3689	66.9	1825	33.1
30–45 mins	2296	14.4	1584	69.0	712	31.0
45–60 mins	827	5.2	538	65.1	289	35.0
> 60 mins	779	4.9	509	65.3	270	34.7
Time to nearest surgery/RT centre by public transport						
Mean (SD)	58.2 (31.9)		58.2 (31.7)		58.1 (32.3)	
Median (IQR)	53 (37–71)		53 (37–71)		52 (37–71)	
< 30 mins	2525	15.8	1702	67.4	823	32.6
30–60 mins	7126	44.6	4707	66.1	2419	34.0
60–90 mins	4023	25.2	2750	68.4	1273	31.6
90–120 mins	1200	7.5	802	66.8	398	33.2
> 120 mins	723	4.5	484	66.9	239	33.1
Missing, invalid/incomplete route	374	2.3	248	66.3	126	33.7

^a^ Percentages in total.

^b^ Percentages in that group.

A total of 10,693 (67%) men received prostatectomy or RT within 12 months of diagnosis. Men receiving radical treatment were younger compared to those not receiving treatment (mean age 68.9 vs. 75.5 years, respectively) with only 18.5% receiving treatment in men aged 80 years or older.

Average travel time to the nearest hospitals was similar for those with and without treatment. Travelling by car required 23.1 min (SD = 18.3) for men receiving treatment and 23.3 min (SD = 19.2) for those who did not receive treatment. For patients travelling by public transport, an average of 58.2 min (SD = 31.7) was needed for the group receiving treatment, compared with 58.1 min (SD = 32.3) for those who did not receive treatment. Treatment rates did not differ according to travel time categories.

### 3.2 Impact of Patient Characteristics and Travel Time on Treatment

Both univariate and multivariable regression models showed a statistically significant impact for age, ethnicity, socioeconomic status and comorbidities (Table 2). Travel time by car to the nearest hospital was not associated with whether men received radical treatment or not.

**TABLE 2:** Unadjusted and adjusted impact of patient characteristics and travel time (by car) on undergoing treatment.

	Unadjusted RR^a^	95% CI^b^	*p* ^c^	Adjusted RR	95% CI	*p*
Age at diagnosis						
< 70	1			1		
70–74	0.94	[0.92–0.96]	< 0.001	0.93	[0.91–0.95]	< 0.001
75–79	0.77	[0.74–0.80]		0.76	[0.73–0.79]	
≥ 80	0.23	[0.20–0.26]		0.23	[0.20–0.26]	
Ethnicity						
White	1			1		
Asian	1.05	[0.98–1.13]	0.02	1.05	[0.98–1.13]	0.001
Black	0.88	[0.79–0.99]		0.88	[0.80–0.96]	
Mixed	0.91	[0.77–1.08]		0.91	[0.77–1.07]	
Other	0.93	[0.84–1.03]		0.89	[0.81–0.97]	
Not stated/Missing	1.02	[0.97–1.07]		0.96	[0.92–1.00]	
IMD (2019) in national quintiles						
1 (least deprived)	1			1		
2	0.98	[0.94–1.01]	< 0.001	0.97	[0.95–1.00]	< 0.001
3	0.97	[0.94–1.01]		0.97	[0.94–1.00]	
4	0.93	[0.89–0.96]		0.93	[0.90–0.96]	
5 (most deprived)	0.92	[0.88–0.96]		0.91	[0.87–0.94]	
Rurality of residence						
Urban (non‐London)	1			1		
Rural	1.02	[1.00–1.05]	0.09	1.00	[0.97–1.02]	0.13
London	0.95	[0.87–1.03]		0.93	[0.86–1.00]	
RCS Charlson score						
0	1			1		
1	0.87	[0.84–0.91]	< 0.001	0.87	[0.84–0.91]	< 0.001
2+	0.66	[0.60–0.72]		0.73	[0.67–0.78]	
Travel time by car						
< 15 mins	1			1		
15–30 mins	1.00	[0.97–1.03]	0.24	0.98	[0.96–1.01]	0.42
30–45 mins	1.03	[0.99–1.08]		1.00	[0.96–1.04]	
45–60 mins	0.97	[0.92–1.04]		0.96	[0.91–1.01]	
> 60 mins	0.98	[0.91–1.05]		0.98	[0.93–1.03]	
No. of observations	15,971			15,971		
No. of groups	123			123		

^a^ Risk ratio obtained by estimating a multilevel GLM model with Poisson family and a log link.

^b^ Robust 95% confidence interval.

^c^ Based on Wald test.

After adjustment, the probability of receiving radical treatment within 12 months of diagnosis was lower for older patients. Compared with men aged < 70, the probability of treatment was 7% lower for those aged 70–74 (adjust Risk Ratio [RR]: 0.93; 95% confidence interval [CI]: 0.91–0.95); 24% lower for those aged 75–79 (adjusted RR: 0.76; 95% CI: 073–0.79) and 77% lower for men older than 80 years (adjusted RR: 0.23; 95% CI: 0.20–0.26). Black men were less likely to receive treatment (adjusted RR: 0.88; 95% CI: 0.80–0.96) as were 'Other' ethnic groups (adjusted RR: 0.89; 95% CI: 0.81–0.97), compared to those with White ethnicity. Treatment was less likely to be delivered to patients of a lower socio‐economic status (adjusted RR: 0.91; 95% CI: 0.87–0.94 for those in the lowest fifth of the national distribution), or those with two or more comorbidities (adjusted RR: 0.73; 95% CI: 0.67–0.78).

### 3.3 Treatment Rate by Patient Characteristics and Travel Time

**FIGURE 2:**
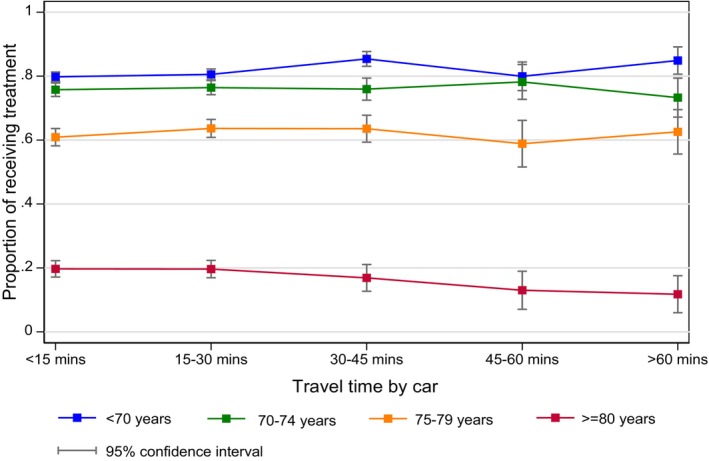
Proportion of patients who received treatment, by age and travel time categories (by car).

We presented crude treatment rates according to patient characteristics and travel time categories by car (Table S2a). Using tests of interaction, we found that in patients aged 80 years or older, the percentage undergoing treatment declined the further away from hospital they lived (Figure 2). We did not observe a similar pattern for other patient characteristics. There was no other statistically significant interaction between travel time by car and patient characteristics (Table S3a). The impact of travel time by public transport on treatment was similar (Table S3b).

## 4 Discussion

This study demonstrates that across the English NHS, approximately one in three patients with high risk and locally advanced prostate cancer do not receive radical treatment. Older patients, those with one or more comorbidities and those living in the most socially deprived areas were less likely to receive radical treatment. There was no association between travel time, either by car or public transport and the likelihood of receiving radical treatment. However, men older than 80 years seemed to be less likely to receive treatment as their travel time increased.

To date, there has been very limited evaluation in the peer‐reviewed literature to understand the impact of the centralisation of cancer services on outcomes of care, particularly with respect to utilisation of evidence based‐treatment. Several studies have sought to understand the impact of centralisation on travel times for care across the population and for vulnerable groups. They largely demonstrate that travel times significantly increased following centralisation, with potentially a disproportionate burden of travel on patients living in rural areas, particularly those from lower socioeconomic groups [4, 5, 22, 23].

Three studies have analysed the impact of travel distance on the likelihood of receiving RT or surgery for prostate cancer. The first study used a US nationwide dataset, demonstrating that as the distance from the patient's residence to the nearest RT facility increased, the proportion of patients receiving RT decreased (53.3% ≤ 5 miles versus 33.8% > 15 miles; *p* < 0.001) [24]. The implications on receipt of surgery were not explicitly explored in this study. Another study in a single US State found no association between travel time and utilisation of RT [25], while the third study from the UK, found that patients were more likely to choose a particular modality depending on which service was closer to their residence [10].

Our study is important as it provides evidence that when accounting for important case mix variables the travel time to a treatment facility is not associated with whether patients receive a radical treatment in the context of the English NHS. We further nuance this by considering whether any association exists according to the type of transport modality, be it public or private transport given the variation in travel times between them. Over the last decade, the number of hospitals providing prostate cancer surgery has been consolidated from 65 to 50. As a result, some patients are facing travel times to their nearest hospital exceeding 90 min in some parts of England [11], but this is not associated with their use of treatment.

The findings are important as they suggest that the current configuration of prostate cancer services in the English NHS relative to the geography of patients is, broadly speaking, ensuring equity of access. Presently, there is significant political pressure to increase and expand access to radiotherapy given the long travel times for some patients to radiotherapy centres, but our analysis suggests that longer travel times are not associated with non‐receipt of treatment. However, the same may not be the case internationally and our study should be used as a basis for understanding the extent to which the configuration of cancer services can directly impact on low rates of treatment utilisation identified in different country contexts and across tumour types [26]. In addition, models are available to support health service planning pre‐implementation by assessing multiple service centralisation scenarios to understand their impact on travel burden, equity in access and outcomes [27].

Similar to previous studies, we do, however, find some inequalities in utilisation of cancer treatments for the most socially deprived, elderly and ethnic minority groups [23]. Potential reasons might relate to differences in personal preferences, healthcare pathways and the interactions among these factors [28]. For elderly men, there is a paucity of data demonstrating that radical treatment, particularly for those over 75 years, given competing risks of mortality is of significant benefit. However, for high‐risk disease, there are additional benefits, including avoidance of lifelong hormonal therapy, increased survival and a reduction in the incidence of metastatic disease. Options to improve assessment of a patient's physical fitness and life expectancy is challenging and likely highly variable. Routine use of comprehensive geriatric assessment has been recommended in addition to the development/increased capacity of onco‐geriatrics services to assess suitability for treatment [29, 30].

Overcoming socioeconomic inequalities is a complex issue to resolve, related not just to improving pathways of care but also to strengthening social determinants of health, which goes beyond what hospitals can influence [31]. Different options have been considered to improve access for lower income and more vulnerable groups, such as the use of pathway navigators [32] that provide continuity of care and focused information around choices. However, again, there is a paucity of data demonstrating how effective such interventions really are. This speaks to the need for more implementation science to design interventions that reflect the broader systemic and service level barriers for vulnerable populations [33]. An intersectionality framework is also another approach to understanding inequities and developing interventions. It specifically considers personal, interpersonal and structural processes, including discrimination, as well as professionals attitudes and behaviours towards minoritised groups [28].

The main strengths of this population‐based study are the large number of patients included and the representativeness of the findings nationally, given that about 95% of UK men with prostate cancer are diagnosed in English NHS hospitals. The accuracy of the routine data we used has also been shown to be consistently high, particularly whether a man received radical treatment. We acknowledge that the period of analysis, April 2019–March 2020, covers the start of the pandemic which may affect utilisation, however the utilisation rates observed are completely consistent when compared to previous national audit results in the years preceding the pandemic [12]. Patient behaviours would have likely changed during the period, with likely increased use of radiotherapy instead of surgery, given that surgical capacity was severely constrained in the 3 months after the pandemic. In addition, some patients who ordinarily would have preferred a radical treatment may have favoured a surveillance approach or prolonged their hormone treatment.

There are several potential determinants of treatment that were not available in our data sources, including patient preferences, and further embedded qualitative work is recommended to further elicit factors associated with treatment preferences and available choices. However, it is unlikely that these factors would significantly impact on the association between travel distance and use of radical treatment beyond those variables accounted for. We didn't use causal inference methods to define our co‐variates, but further work using directed acyclic graphs, could be considered to provide further robustness to the analysis. Finally, we have undertaken the analysis focusing on travel times by car or by public transport however, information is not available on the mode of transport used by the patient. In 2021, approximately 25% of households in England did not have a car, with non‐car ownership concentrated in the lowest income households [34, 35]. We found in a previous study that travel times by public transport were on average twice as long compared to travel times by car and in some parts of England, particularly rural areas, reliance on public transport was associated with a further 2 h compared to those with car access [11]. Therefore, future studies should explore the use of the information on car ownership or public transport accessibility to account for this limitation.

## 5 Conclusion

This national population‐based study of approximately 16,000 men finds that one in three men diagnosed with high risk localised or locally advanced prostate cancer do not receive curative treatment. In the era of increasing cancer service centralisation, we find that travel times by car or public transport are not associated with the likelihood of receiving a treatment, suggesting further increases in capacity will not improve utilisation rates. Inequalities in treatment use were evident, particularly for men aged 70 or over and those living in the most socially deprived areas. Interventions and capacity to increase treatment utilisation should be targeted in this group.

## Author Contributions

**Lu Han:** data curation (equal), formal analysis (lead), visualization (lead), writing – original draft (equal), writing – review and editing (equal). **Emily Mayne:** data curation (equal), writing – review and editing (equal). **Joanna Dodkins:** writing – review and editing (equal). **Richard Sullivan:** writing – review and editing (equal). **Adrian Cook:** writing – review and editing (equal). **Matthew Parry:** writing – review and editing (equal). **Julie Nossiter:** writing – review and editing (equal). **Thomas E. Cowling:** writing – review and editing (equal). **Alison Tree:** writing – review and editing (equal). **Noel Clarke:** writing – review and editing (equal). **Jan van der Meulen:** conceptualization (equal), methodology (equal), writing – review and editing (equal). **Ajay Aggarwal:** conceptualization (equal), funding acquisition (lead), methodology (equal), writing – original draft (equal), writing – review and editing (equal).

## Ethics Statement

Ethics approval for use of secondary anonymised patient level datasets for these analyses was received from the NHS Research Ethics Committee on 06.01.2020, reference: 20/WA/0161. Informed consent was not required for use of this information.

## Conflicts of Interest

The authors declare no conflicts of interest.

## Supporting information


Appendix S1.

## Data Availability

This study was based on English national cancer registry data. We do not own these data and hence are not permitted to share them in the original form. The data are available from the NHS Digital's Data Access Request Service. For access, please email data.applications@nhsdigital.nhs.uk.
