# Supplementary material for: Is Centralisation of Cancer Services Associated With Under‐Treatment of Patients With High‐Risk Prostate Cancer?—A National Population‐Based Study
Source: Cancer Med. 2024 Nov 11;13(21):e70403. doi: 10.1002/cam4.70403 (PMC11551782; doi:10.1002/cam4.70403)
Supplement: Supplementary file 1 — Appendix S1. [file CAM4-13-e70403-s001.docx]

APPENDIX

**Appendix Figure 1. Patient exclusion flow chart.**


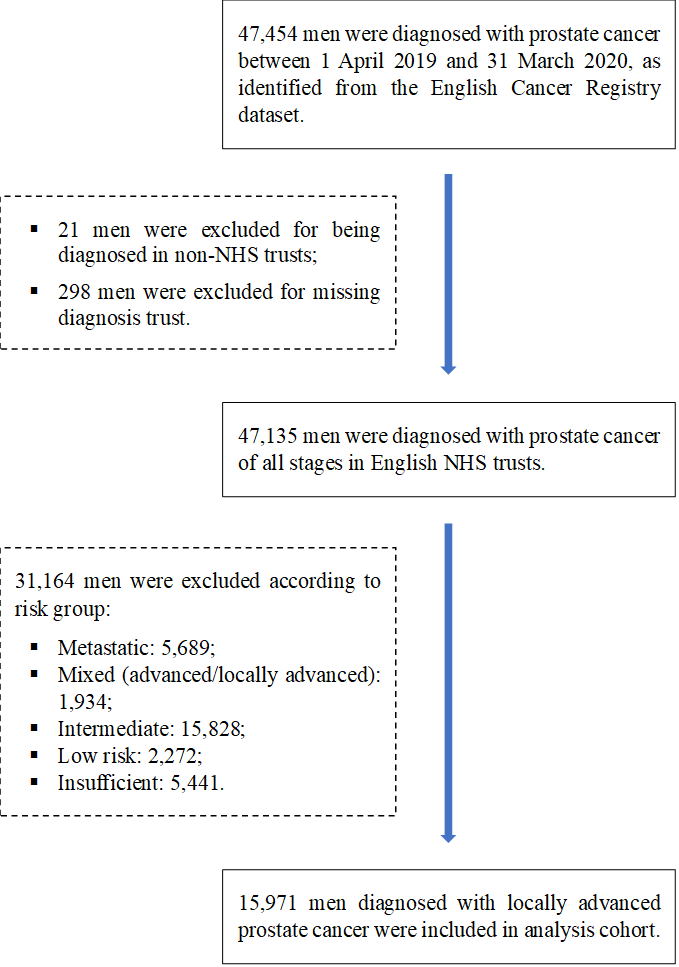


**Appendix Table 1. Unadjusted and adjusted impact of patient characteristics and travel time (by public transport) on undergoing treatment.**

|  | **Unadjusted**  **RR^a^** | **95% CI^b^** | ***P* value^c^** | **Adjusted RR** | **95% CI** | ***P* value** |
| --- | --- | --- | --- | --- | --- | --- |
| **Age at diagnosis** | | | | | | |
| <70 | 1 |  |  | 1 |  |  |
| 70-74 | 0.94 | [0.92 - 0.96] | **<0.001** | 0.93 | [0.91 - 0.95] | **<0.001** |
| 75-79 | 0.77 | [0.74 - 0.80] |  | 0.76 | [0.73 - 0.79] |  |
| >=80 | 0.23 | [0.20 - 0.26] |  | 0.23 | [0.20 - 0.26] |  |
| **Ethnicity** | | | | | | |
| White | 1 |  |  | 1 |  |  |
| Asian | 1.05 | [0.98 - 1.13] | **0.02** | 1.05 | [0.98 - 1.13] | **<0.001** |
| Black | 0.88 | [0.79 - 0.99] |  | 0.88 | [0.80 - 0.96] |  |
| Mixed | 0.91 | [0.77 - 1.08] |  | 0.91 | [0.77 - 1.07] |  |
| Other | 0.93 | [0.84 - 1.03] |  | 0.89 | [0.81 - 0.97] |  |
| Not stated/Missing | 1.02 | [0.97 - 1.07] |  | 0.96 | [0.92 - 1.00] |  |
| **IMD (2019) in national quintiles** | | | | | | |
| 1 (least deprived) | 1 |  |  | 1 |  |  |
| 2 | 0.98 | [0.94 - 1.01] | **<0.001** | 0.97 | [0.95 - 1.00] | **<0.001** |
| 3 | 0.97 | [0.94 - 1.01] |  | 0.97 | [0.95 - 1.00] |  |
| 4 | 0.93 | [0.89 - 0.96] |  | 0.93 | [0.90 - 0.96] |  |
| 5 (most deprived) | 0.92 | [0.88 - 0.96] |  | 0.91 | [0.87 - 0.94] |  |
| **Rurality of residence** | | | | | | |
| Urban (non-London) | 1 |  |  | 1 |  |  |
| Rural | 1.02 | [1.00 - 1.05] | 0.09 | 1.00 | [0.98 - 1.02] | 0.16 |
| London | 0.95 | [0.87 - 1.03] |  | 0.93 | [0.87 - 1.00] |  |
| **RCS Charlson score** | | | | | | |
| 0 | 1 |  |  | 1 |  |  |
| 1 | 0.87 | [0.84 - 0.91] | **<0.001** | 0.87 | [0.84 - 0.91] | **<0.001** |
| 2+ | 0.66 | [0.60 - 0.72] |  | 0.73 | [0.67 - 0.78] |  |
| **Travel time by public transport** | | | | | | |
| <30 mins | 1 |  |  | 1 |  |  |
| 30-60 mins | 0.98 | [0.95 - 1.01] | 0.44 | 0.97 | [0.94 - 1.00] | 0.22 |
| 60-90 mins | 1.01 | [0.97 - 1.05] |  | 0.98 | [0.94 - 1.02] |  |
| 90-120 mins | 0.99 | [0.94 - 1.04] |  | 0.95 | [0.90 - 1.01] |  |
| >120 mins | 0.99 | [0.92 - 1.06] |  | 0.95 | [0.89 - 1.00] |  |
| Missing time/invalid route | 0.99 | [0.87 - 1.12] |  | 0.95 | [0.86 - 1.05] |  |
|  |  |  |  |  |  |  |
| **No. of observations** | 15,971 |  |  | 15,971 |  |  |
| **No. of groups** | 123 |  |  | 123 |  |  |
| a. Risk ratio obtained by estimating a multilevel GLM model with Poisson family and a log link.  b. Robust 95% confidence interval.  c. Based on Wald test. | | | | | | |

**Appendix Table 2a. Crude treatment rate by patient characteristics and travel time categories (by car).**

|  | **<15 mins** | | **15-30 mins** | | **30-45 mins** | | **45-60 mins** | | **>60 mins** | |
| --- | --- | --- | --- | --- | --- | --- | --- | --- | --- | --- |
|  | **Total** | **Treated** | **Total** | **Treated** | **Total** | **Treated** | **Total** | **Treated** | **Total** | **Treated** |
| **Age at diagnosis** | | | | | | | | | | |
| <70 | 2,799 | 2,233 (79.8%) | 2,148 | 1,730 (80.5%) | 896 | 765 (85.4%) | 309 | 247 (79.9%) | 271 | 230 (84.9%) |
| 70-74 | 1,587 | 1,202 (75.7%) | 1,411 | 1,078 (76.4%) | 590 | 448 (75.9%) | 220 | 172 (78.2%) | 202 | 148 (73.3%) |
| 75-79 | 1,240 | 755 (60.9%) | 1,130 | 719 (63.6%) | 502 | 319 (63.5%) | 175 | 103 (58.9%) | 187 | 117 (62.6%) |
| >=80 | 929 | 183 (19.7%) | 825 | 162 (19.6%) | 308 | 52  (16.9%) | 123 | 16 (13.0%) | 119 | 14 (11.8%) |
| **Ethnicity** | | | | | | | | | | |
| White | 5,383 | 3,613 (67.1%) | 4,890 | 3,279 (67.1%) | 2,065 | 1,420 (68.8%) | 743 | 480 (64.6%) | 691 | 452 (65.4%) |
| Asian | 204 | 143  (70.1%) | 54 | 38  (70.4%) | 8 | 7  (87.5%) | 0 | 0  (.%) | 1 | 1  (100%) |
| Black | 321 | 186  (57.9%) | 81 | 47  (58.0%) | 11 | 9  (81.8%) | 3 | 3  (100%) | 1 | 1  (100%) |
| Mixed | 48 | 30  (62.5%) | 16 | 8  (50.0%) | 4 | 3  (75.0%) | 3 | 2  (66.7%) | 2 | 2  (100%) |
| Other | 137 | 82  (59.9%) | 59 | 35  (59.3%) | 14 | 12  (85.7%) | 10 | 8  (80.0%) | 6 | 4  (66.7%) |
| Not stated/Missing | 462 | 319  (69.0%) | 414 | 282  (68.1%) | 194 | 133  (68.6%) | 68 | 45  (66.2%) | 78 | 49  (62.8%) |
| **IMD (2019) in national quintiles** | | | | | | | | | | |
| 1 (least deprived) | 1,303 | 902  (69.2%) | 1,739 | 1,197 (68.8%) | 578 | 413  (71.5%) | 116 | 76  (65.5%) | 82 | 60  (73.2%) |
| 2 | 1,410 | 960  (68.1%) | 1,337 | 900  (67.3%) | 753 | 511  (67.9%) | 273 | 188 (68.9%) | 209 | 136 (65.1%) |
| 3 | 1,303 | 902  (69.2%) | 1,097 | 740  (67.5%) | 508 | 356  (70.1%) | 263 | 156 (59.3%) | 249 | 155 (62.2%) |
| 4 | 1,225 | 783  (63.9%) | 822 | 515  (62.7%) | 308 | 206  (66.9%) | 135 | 92  (68.1%) | 170 | 110 (64.7%) |
| 5 (most deprived) | 1,314 | 826  (62.9%) | 519 | 337  (64.9%) | 149 | 98  (65.8%) | 40 | 26  (65.0%) | 69 | 48  (69.6%) |
| **RCS Charlson score** | | | | | | | | | | |
| 0 | 5,515 | 3,802 (68.9%) | 4,794 | 3,272 (68.3%) | 2,018 | 1,431 (70.9%) | 724 | 477 (65.9%) | 670 | 453 (67.6%) |
| 1 | 738 | 444  (60.2%) | 527 | 319  (60.5%) | 199 | 118  (59.3%) | 76 | 46  (60.5%) | 83 | 47  (56.6%) |
| 2+ | 302 | 127  (42.1%) | 193 | 98  (50.8%) | 79 | 35  (44.3%) | 27 | 15  (55.6%) | 26 | 9  (34.6%) |
| **Rurality of residence** | | | | | | | | | | |
| Rural | 362 | 256  (70.7%) | 1,618 | 1,110 (68.6%) | 1,138 | 791  (69.5%) | 577 | 377 (65.3%) | 460 | 305 (66.3%) |
| Urban (non-London) | 4,904 | 3,286 (67.0%) | 3,714 | 2,474 (66.6%) | 1,158 | 793  (68.5%) | 250 | 161 (64.4%) | 319 | 204 (63.9%) |
| London | 1,289 | 831  (64.5%) | 182 | 105  (57.7%) | 0 | 0  (.%) | 0 | 0  (.%) | 0 | 0  (.%) |

**Appendix Table 2b. Crude treatment rate by patient characteristics and travel time categories (by public transport).**

|  | **<30 mins** | | **30-60 mins** | | **60-90 mins** | | **90-120 mins** | | **>120 mins** | | **Missing time/invalid  route** | |
| --- | --- | --- | --- | --- | --- | --- | --- | --- | --- | --- | --- | --- |
|  | **Total** | **Treated** | **Total** | **Treated** | **Total** | **Treated** | **Total** | **Treated** | **Total** | **Treated** | **Total** | **Treated** |
| **Age at diagnosis** | | | | | | | | | | | | |
| <70 | 1,090 | 890 (81.7%) | 2,898 | 2,292 (79.1%) | 1,567 | 1,308 (83.5%) | 445 | 367 (82.5%) | 286 | 231 (80.8%) | 137 | 117 (85.4%) |
| 70-74 | 588 | 451 (76.7%) | 1,772 | 1,333 (75.2%) | 1,034 | 804 (77.8%) | 321 | 236 (73.5%) | 192 | 145 (75.5%) | 103 | 79 (76.7%) |
| 75-79 | 469 | 285 (60.8%) | 1,402 | 873 (62.3%) | 862 | 542 (62.9%) | 273 | 173 (63.4%) | 153 | 96 (62.7%) | 75 | 44 (58.7%) |
| >=80 | 378 | 76 (20.1%) | 1,054 | 209 (19.8%) | 560 | 96 (17.1%) | 161 | 26 (16.1%) | 92 | 12 (13.0%) | 59 | 8 (13.6%) |
| **Ethnicity** | | | | | | | | | | | | |
| White | 2,100 | 1,417 (67.5%) | 6,017 | 4,003 (66.5%) | 3,602 | 2,457 (68.2%) | 1,063 | 712 (67.0%) | 639 | 420 (65.7%) | 351 | 235 (67.0%) |
| Asian | 75 | 58 (77.3%) | 161 | 108 (67.1%) | 25 | 18 (72.0%) | 5 | 4 (80.0%) | 1 | 1 (100%) | 0 | 0 (.%) |
| Black | 106 | 58 (54.7%) | 264 | 156 (59.1%) | 41 | 27 (65.9%) | 4 | 3 (75.0%) | 2 | 2 (100%) | 0 | 0 (.%) |
| Mixed | 22 | 14 (63.6%) | 33 | 19 (57.6%) | 14 | 8 (57.1%) | 2 | 2 (100%) | 1 | 1 (100%) | 1 | 1 (100%) |
| Other | 46 | 30 (65.2%) | 129 | 73 (56.6%) | 33 | 23 (69.7%) | 11 | 10 (90.9%) | 6 | 5 (83.3%) | 1 | 0 (0%) |
| Not stated/Missing | 176 | 125 (71.0%) | 522 | 348 (66.7%) | 308 | 217 (70.5%) | 115 | 71 (61.7%) | 74 | 55 (74.3%) | 21 | 12 (57.1%) |
| **IMD (2019) in national quintiles** | | | | | | | | | | | | |
| 1 | 474 | 328 (69.2%) | 1,747 | 1,187 (67.9%) | 1,098 | 784 (71.4%) | 294 | 208 (70.7%) | 104 | 72 (69.2%) | 101 | 69 (68.3%) |
| 2 | 485 | 322 (66.4%) | 1,666 | 1,123 (67.4%) | 1,052 | 723 (68.7%) | 396 | 266 (67.2%) | 241 | 167 (69.3%) | 142 | 94 (66.2%) |
| 3 | 530 | 386 (72.8%) | 1,401 | 941 (67.2%) | 876 | 597 (68.2%) | 306 | 194 (63.4%) | 227 | 137 (60.4%) | 80 | 54 (67.5%) |
| 4 | 492 | 321 (65.2%) | 1,242 | 777 (62.6%) | 626 | 407 (65.0%) | 141 | 94 (66.7%) | 115 | 81 (70.4%) | 44 | 26 (59.1%) |
| 5 | 544 | 345 (63.4%) | 1,070 | 679 (63.5%) | 371 | 239 (64.4%) | 63 | 40 (63.5%) | 36 | 27 (75.0%) | 7 | 5 (71.4%) |
| **RCS Charlson score** | | | | | | | | | | | | |
| 0 | 2,095 | 1,470 (70.2%) | 6,106 | 4,146 (67.9%) | 3,520 | 2,455 (69.7%) | 1,045 | 712 (68.1%) | 631 | 429 (68.0%) | 324 | 223 (68.8%) |
| 1 | 299 | 177 (59.2%) | 747 | 439 (58.8%) | 355 | 227 (63.9%) | 111 | 65 (58.6%) | 65 | 41 (63.1%) | 46 | 25 (54.3%) |
| 2+ | 131 | 55 (42.0%) | 273 | 122 (44.7%) | 148 | 68 (45.9%) | 44 | 25 (56.8%) | 27 | 14 (51.9%) | 4 | 0 (0%) |
| **Rurality of residence** | | | | | | | | | | | | |
| Rural | 68 | 43 (63.2%) | 959 | 654 (68.2%) | 1,409 | 970 (68.8%) | 838 | 577 (68.9%) | 590 | 397 (67.3%) | 291 | 198 (68.0%) |
| Urban (non-London) | 2,029 | 1,370 (67.5%) | 5,149 | 3,425 (66.5%) | 2,589 | 1,761 (68.0%) | 362 | 225 (62.2%) | 133 | 87 (65.4%) | 83 | 50 (60.2%) |
| London | 428 | 289 (67.5%) | 1,018 | 628 (61.7%) | 25 | 19 (76.0%) | 0 | 0 (.%) | 0 | 0 (.%) | 0 | 0 (.%) |

**Appendix Table 3a. Adjusted impact of patient characteristics, travel time (by car) and interaction effects between patient characteristics and travel time, on undergoing treatment.**

|  | **Adjusted RR^a^** | **95% CI^b^** | ***p* value^c^** | **Adjusted RR** | **95% CI** | ***p* value** | **Adjusted RR** | **95% CI** | ***p* value** | **Adjusted RR** | **95% CI** | ***p* value** | **Adjusted RR** | **95% CI** | ***p* value** |
| --- | --- | --- | --- | --- | --- | --- | --- | --- | --- | --- | --- | --- | --- | --- | --- |
| **Age at diagnosis** | | | | | | | | | | | | | | | |
| <80 | 1 |  |  | 1 |  |  | 1 |  |  | 1 |  |  | 1 |  |  |
| >=80 | 0.26 | [0.23-0.30] | <0.001 | 0.25 | [0.21-0.28] | <0.001 | 0.25 | [0.21-0.28] | <0001 | 0.25 | [0.21-0.28] | <0.001 | 0.25 | [0.21-0.28] | <0.001 |
| **Ethnicity** | | | | | | | | | | | | | | | |
| White | 1 |  |  | 1 |  |  | 1 |  |  | 1 |  |  | 1 |  |  |
| Non-White | 0.93 | [0.87-0.99] | 0.03 | 0.91 | [0.86-0.97] | 0.02 | 0.93 | [0.87-0.99] | 0.03 | 0.93 | [0.87-0.99] | 0.03 | 0.93 | [0.87-0.99] | 0.03 |
| Not stated/missing | 0.97 | [0.93-1.02] |  | 0.98 | [0.93-1.03] |  | 0.97 | [0.93-1.02] |  | 0.97 | [0.93-1.02] |  | 0.97 | [0.93-1.02] |  |
| **IMD (2019) in national quintiles** | | | | | | | | | | | | | | | |
| 1-2 | 1 |  |  | 1 |  |  | 1 |  |  | 1 |  |  | 1 |  |  |
| 3-5 (most deprived) | 0.96 | [0.94-0.98] | <0.001 | 0.96 | [0.94-0.98] | <0.001 | 0.96 | [0.93-0.98] | <0.001 | 0.96 | [0.94-0.98] | <0.001 | 0.96 | [0.94-0.98] | <0.001 |
| **Rurality of residence** | | | | | | | | | | | | | | | |
| Urban (incl. London) | 1 |  |  | 1 |  |  | 1 |  |  | 1 |  |  | 1 |  |  |
| Rural | 1.00 | [0.98-1.02] | 0.99 | 1.00 | [0.98-1.02] | 0.99 | 1.00 | [0.98-1.02] | 0.98 | 1.01 | [0.98-1.04] | 0.52 | 1.00 | [0.98-1.02] | 0.97 |
| **Charlson comorbidities** | | | | | | | | | | | | | | | |
| Without comorbidity | 1 |  |  | 1 |  |  | 1 |  |  | 1 |  |  | 1 |  |  |
| With comorbidity | 0.83 | [0.80-0.86] | <0.001 | 0.83 | [0.80-0.86] | <0.001 | 0.83 | [0.80-0.86] | <0.001 | 0.83 | [0.80-0.86] | <0.001 | 0.84 | [0.80-0.87] | <0.001 |
| **Travel time by car** | | | | | | | | | | | | | | | |
| <30 mins | 1 |  |  | 1 |  |  | 1 |  |  | 1 |  |  | 1 |  |  |
| 30-60 mins | 1.01 | [0.98-1.04] | 0.83 | 1.00 | [0.96-1.03] | 0.86 | 1.00 | [0.96-1.04] | 0.89 | 1.01 | [0.97-1.07] | 0.46 | 1.00 | [0.97-1.04] | 0.95 |
| >60 mins | 1.00 | [0.95-1.05] |  | 0.98 | [0.93-1.04] |  | 0.98 | [0.91-1.06] |  | 0.97 | [0.92-1.03] |  | 0.99 | [0.94-1.05] |  |
| **Age * Travel time by car** | | | | | | | | | | | | | | | |
| >=80 * 30-60 mins | 0.79 | [0.57-1.08] | 0.27 |  |  |  |  |  |  |  |  |  |  |  |  |
| >=80 * >60 mins | 0.59 | [0.27-1.30] |  |  |  |  |  |  |  |  |  |  |  |  |  |
| **Ethnicity * Travel time by car** | | | | | | | | | | | | | | | |
| Non-White  * 30-60 mins |  |  |  | 1.21 | [1.06-1.38] | 0.06 |  |  |  |  |  |  |  |  |  |
| Non-White  * >60 mins |  |  |  | 1.18 | [0.84-1.68] |  |  |  |  |  |  |  |  |  |  |
| Not stated/missing  * 30-60 mins |  |  |  | 1.00 | [0.91-1.10] |  |  |  |  |  |  |  |  |  |  |
| Not stated/missing * >60 mins |  |  |  | 0.95 | [0.80-1.12] |  |  |  |  |  |  |  |  |  |  |
| **IMD (2019) in national quintiles * Travel time by car** | | | | | | | | | | | | | | | |
| 3-5 * 30-60 mins |  |  |  |  |  |  | 1.01 | [0.96-1.06] | 0.95 |  |  |  |  |  |  |
| 3-5 * >60 mins |  |  |  |  |  |  | 1.00 | [0.93-1.09] |  |  |  |  |  |  |  |
| **Rurality of residence * Travel time by car** | | | | | | | | | | | | | | | |
| Rural * 30-60 mins |  |  |  |  |  |  |  |  |  | 0.97 | [0.91-1.03] | 0.58 |  |  |  |
| Rural * >60 mins |  |  |  |  |  |  |  |  |  | 1.01 | [0.93-1.09] |  |  |  |  |
| **Charlson comorbidities * Travel time by car** | | | | | | | | | | | | | | | |
| With comorbidity  * 30-60 mins |  |  |  |  |  |  |  |  |  |  |  |  | 0.99 | [0.88-1.11] | 0.43 |
| With comorbidity * >60 mins |  |  |  |  |  |  |  |  |  |  |  |  | 0.91 | [0.79-1.05] |  |
|  |  |  |  |  |  |  |  |  |  |  |  |  |  |  |  |
| **No. of observations** | 15,971 |  |  | 15,971 |  |  | 15,971 |  |  | 15,971 |  |  | 15,971 |  |  |
| **No. of groups** | 123 |  |  | 123 |  |  | 123 |  |  | 123 |  |  | 123 |  |  |
| a. Risk ratio obtained by estimating a multilevel GLM model with Poisson family and a log link.  b. Robust 95% confidence interval.  c. Combined *p* value obtained using Wald test. | | | | | | | | | | | | | | | |

**Appendix Table 3b. Adjusted impact of patient characteristics, travel time (by public transport) and interaction effects between patient characteristics and travel time, on undergoing treatment.**

|  | **Adjusted RR^a^** | **95% CI^b^** | ***p* value^c^** | **Adjusted RR** | **95% CI** | ***p* value** | **Adjusted RR** | **95% CI** | ***p* value** | **Adjusted RR** | **95% CI** | ***p* value** | **Adjusted RR** | **95% CI** | ***p* value** |
| --- | --- | --- | --- | --- | --- | --- | --- | --- | --- | --- | --- | --- | --- | --- | --- |
| **Age at diagnosis** | | | | | | | | | | | | | | | |
| <80 | 1 |  |  | 1 |  |  | 1 |  |  | 1 |  |  | 1 |  |  |
| >=80 | 0.27 | [0.23-0.31] | <0.001 | 0.25 | [0.21-0.28] | <0.001 | 0.25 | [0.21-0.28] | <0.001 | 0.25 | [0.21-0.28] | <0.001 | 0.25 | [0.21-0.28] | <0.001 |
| **Ethnicity** | | | | | | | | | | | | | | | |
| White | 1 |  |  | 1 |  |  | 1 |  |  | 1 |  |  | 1 |  |  |
| Non-White | 0.93 | [0.87-0.99] | 0.03 | 0.91 | [0.85-0.98] | 0.02 | 0.93 | [0.87-0.98] | 0.03 | 0.93 | [0.87-0.98] | 0.03 | 0.93 | [0.87-0.99] | 0.03 |
| Not stated/missing | 0.97 | [0.93-1.02] |  | 0.96 | [0.91-1.01] |  | 0.97 | [0.93-1.02] |  | 0.97 | [0.93-1.02] |  | 0.97 | [0.93-1.02] |  |
| **IMD (2019) in national quintiles** | | | | | | | | | | | | | | | |
| 1-2 | 1 |  |  | 1 |  |  | 1 |  |  | 1 |  |  | 1 |  |  |
| 3-5 (most deprived) | 0.96 | [0.94-0.98] | <0.001 | 0.96 | [0.94-0.98] | <0.001 | 0.96 | [0.93-0.99] | 0.004 | 0.96 | [0.94-0.98] | <0.001 | 0.96 | [0.94-0.98] | <0.001 |
| **Rurality of residence** | | | | | | | | | | | | | | | |
| Urban (incl. London) | 1 |  |  | 1 |  |  | 1 |  |  | 1 |  |  | 1 |  |  |
| Rural | 1.00 | [0.98-1.03] | 0.86 | 1.00 | [0.98-1.03] | 0.84 | 1.00 | [0.98-1.02] | 0.90 | 0.99 | [0.95-1.03] | 0.56 | 1.00 | [0.98-1.03] | 0.84 |
| **Charlson comorbidities** | | | | | | | | | | | | | | | |
| Without comorbidity | 1 |  |  | 1 |  |  | 1 |  |  | 1 |  |  | 1 |  |  |
| With comorbidity | 0.83 | [0.80-0.86] | <0.001 | 0.83 | [0.80-0.86] | <0.001 | 0.83 | [0.80-0.86] | <0.001 | 0.83 | [0.80-0.86] | <0.001 | 0.82 | [0.78-0.86] | <0.001 |
| **Travel time by public transport** | | | | | | | | | | | | | | | |
| <60 mins | 1 |  |  | 1 |  |  | 1 |  |  | 1 |  |  | 1 |  |  |
| 60-120 mins | 1.01 | [0.98-1.04] | 0.77 | 1.00 | [0.97-1.03] | 0.47 | 1.01 | [0.98-1.04] | 0.55 | 1.00 | [0.97-1.03] | 0.67 | 1.00 | [0.97-1.03] | 0.61 |
| >120 mins | 0.99 | [0.93-1.05] |  | 0.96 | [0.90-1.01] |  | 0.97 | [0.90-1.04] |  | 0.99 | [0.86-1.15] |  | 0.96 | [0.91-1.02] |  |
| Missing/invalid route | 1.00 | [0.93-1.09] |  | 0.99 | [0.89-1.11] |  | 0.98 | [0.87-1.09] |  | 0.94 | [0.84-1.04] |  | 1.00 | [0.90-1.11] |  |
| **Age * Travel time by public transport** | | | | | | | | | | | | | | | |
| >=80 * 60-120 mins | 0.83 | [0.64-1.07] | 0.34 |  |  |  |  |  |  |  |  |  |  |  |  |
| >=80 * >120 mins | 0.66 | [0.39-1.12] |  |  |  |  |  |  |  |  |  |  |  |  |  |
| >=80 * Missing /invalid route | 0.66 | [0.27-1.63] |  |  |  |  |  |  |  |  |  |  |  |  |  |
| **Ethnicity * Travel time by public transport** | | | | | | | | | | | | | | | |
| Non-White *  60-120 mins |  |  |  | 1.07 | [0.93-1.22] | 0.02 |  |  |  |  |  |  |  |  |  |
| Non-White *  >120 mins |  |  |  | 1.32 | [1.13-1.53] |  |  |  |  |  |  |  |  |  |  |
| Non-White *  Missing /invalid route |  |  |  | 0.79 | [0.22-2.84] |  |  |  |  |  |  |  |  |  |  |
| Not stated/missing * 60-120 mins |  |  |  | 1.02 | [0.94-1.12] |  |  |  |  |  |  |  |  |  |  |
| Not stated/missing *  >120 mins |  |  |  | 1.17 | [0.98-1.41] |  |  |  |  |  |  |  |  |  |  |
| Not stated/missing * Missing /invalid route |  |  |  | 0.92 | [0.61-1.37] |  |  |  |  |  |  |  |  |  |  |
| **IMD (2019) in national quintiles * Travel time by public transport** | | | | | | | | | | | | | | | |
| 3-5 * 60-120 mins |  |  |  |  |  |  | 0.99 | [0.95-1.03] | 0.67 |  |  |  |  |  |  |
| 3-5 * >120 mins |  |  |  |  |  |  | 1.02 | [0.93-1.12] |  |  |  |  |  |  |  |
| 3-5 * Missing /invalid route |  |  |  |  |  |  | 1.04 | [0.94-1.15] |  |  |  |  |  |  |  |
| **Rurality of residence * Travel time by public transport** | | | | | | | | | | | | | | | |
| Rural * 60-120 mins |  |  |  |  |  |  |  |  |  | 1.02 | [0.97-1.07] | 0.62 |  |  |  |
| Rural * >120 mins |  |  |  |  |  |  |  |  |  | 0.99 | [0.85-1.16] |  |  |  |  |
| Rural * Missing /invalid route |  |  |  |  |  |  |  |  |  | 1.08 | [0.95-1.23] |  |  |  |  |
| **Charlson comorbidities * Travel time by public transport** | | | | | | | | | | | | | | | |
| With comorbidity *  60-120 mins |  |  |  |  |  |  |  |  |  |  |  |  | 1.03 | [0.94-1.13] | 0.33 |
| With comorbidity *  >120 mins |  |  |  |  |  |  |  |  |  |  |  |  | 1.11 | [0.96-1.28] |  |
| With comorbidity * Missing /invalid route |  |  |  |  |  |  |  |  |  |  |  |  | 0.87 | [0.67-1.13] |  |
|  |  |  |  |  |  |  |  |  |  |  |  |  |  |  |  |
| **No. of observations** | 15,971 |  |  | 15,971 |  |  | 15,971 |  |  | 15,971 |  |  | 15,971 |  |  |
| **No. of groups** | 123 |  |  | 123 |  |  | 123 |  |  | 123 |  |  | 123 |  |  |
| a. Risk ratio obtained by estimating a multilevel GLM model with Poisson family and a log link.  b. Robust 95% confidence interval.  c. Combined *p* value obtained using Wald test. | | | | | | | | | | | | | | | |
